# Smartphone-Integrated Electrochemical Devices for Contaminant Monitoring in Agriculture and Food: A Review

**DOI:** 10.3390/bios15090574

**Published:** 2025-09-02

**Authors:** Sumeyra Savas, Seyed Mohammad Taghi Gharibzahedi

**Affiliations:** 1 Biosensors and Biotechnology Laboratory (BBL), Medical School, Bandırma Onyedi Eylül University, 10200 Bandırma, Balıkesir, Türkiye; 2 Institute of Materials Science, Faculty of Engineering, Kiel University, 24143 Kiel, Germany; smg@tf.uni-kiel.de

**Keywords:** lab on a chip, electrochemical sensors, smartphone-based detection, food contaminants, sustainable agriculture, agricultural surveillance, pesticide residue analysis, food supply chain, point of care testing, microfluidics

## Abstract

Recent progress in microfluidic technologies has led to the development of compact and highly efficient electrochemical platforms, including lab-on-a-chip (LoC) systems, that integrate multiple testing functions into a single, portable device. Combined with smartphone-based electrochemical devices, these systems enable rapid and accurate on-site detection of food contaminants, including pesticides, heavy metals, pathogens, and chemical additives at farms, markets, and processing facilities, significantly reducing the need for traditional laboratories. Smartphones improve the performance of these platforms by providing computational power, wireless connectivity, and high-resolution imaging, making them ideal for in-field food safety testing with minimal sample and reagent requirements. At the core of these systems are electrochemical biosensors, which convert specific biochemical reactions into electrical signals, ensuring highly sensitive and selective detection. Advanced nanomaterials and integration with Internet of Things (IoT) technologies have further improved performance, delivering cost-effective, user-friendly food monitoring solutions that meet regulatory safety and quality standards. Analytical techniques such as voltammetry, amperometry, and impedance spectroscopy increase accuracy even in complex food samples. Moreover, low-cost engineering, artificial intelligence (AI), and nanotechnology enhance the sensitivity, affordability, and data analysis capabilities of smartphone-integrated electrochemical devices, facilitating their deployment for on-site monitoring of food and agricultural contaminants. This review explains how these technologies address global food safety challenges through rapid, reliable, and portable detection, supporting food quality, sustainability, and public health.

## 1. Introduction

The rise of industrial agriculture and modern food production has greatly increased the risk of introducing hazardous substances into food and the environment. These contaminants come from many sources, including pesticides, veterinary drugs, heavy metals, persistent organic pollutants, pathogenic microorganisms, and by-products of agricultural and industrial activities [1,2,3]. These substances can enter the food chain through soil, water, feed, or during processing and packaging. They tend to bioaccumulate in animals and humans, leading to both acute and chronic health effects [1,4]. Foodborne pathogens, such as *Escherichia coli*, *Salmonella*, and *Listeria*, as well as chemical residues from fertilizers and pesticides, represent serious threats to food safety and public health [5,6]. Climate change, intensive farming, and globalization of the food supply chain further intensify these risks, making contaminants widespread and difficult to control using conventional methods alone [2].

Ensuring safe, high-quality food is a critical global challenge, as contamination not only threatens human health but also causes significant economic and social costs [6,7,8,9,10]. Effective monitoring of contaminants is essential to prevent foodborne illnesses, protect consumers, and maintain confidence in the food supply [1,4]. Although traditional laboratory-based detection methods are sensitive and reliable, they are often too time-consuming, expensive, and impractical for routine or on-site use [4,5]. Therefore, developing rapid, portable, and cost-effective detection tools, such as biosensors and electrochemical devices, is crucial for real-time surveillance of biological and chemical hazards in food and agricultural products [2,3,4,5,11,12,13,14,15]. Such monitoring enables timely interventions, reduces health risks, ensures regulatory compliance, and supports the sustainability and security of the global food system [1,6,11,12,13,16,17].

Biosensors are analytical devices that use biological components to recognize specific substances or physiological changes and translate them in measurable signals through an electronic system [18]. These systems play a key role in detecting, recording, and transmitting information about the chemical or biological composition of their surroundings [9,11,13]. Unlike conventional bioanalytical techniques, which typically rely on the reactions between a sample and chemical reagents in a liquid phase, biosensors show a distinct approach by functioning without the need for externally added reagents during measurement. The necessary reactive components in biosensing systems are directly immobilized on the transducer surface, eliminating the need for external reagent addition or complex sample pretreatment during analysis [17,19].

A variety of biological recognition elements can be utilized in these systems, including antibodies, aptamers, nucleic acids, enzymes, whole cells, tissues, and molecularly imprinted polymers (MIPs). Choosing an appropriate recognition element depends on both the identity of the target analyte and the specific parameters of the sensing method [20]. Enzymes, as structurally stable and highly specific protein based biocatalysts, are particularly convenient for immobilization on receptor surfaces. This property has made them important components in the design of biosensing platforms [21]. Antibodies are immunoglobulin proteins produced by the immune system that exhibit high specificity and strong affinity toward their target molecules [22]. In contrast, aptamers are short, single-stranded DNA or RNA oligonucleotides synthetically designed to bind selectively and tightly to specific targets [8]. Due to their high binding affinity, chemical stability, and ease of modification, aptamers have emerged as promising molecular recognition elements in biosensor development, offering a viable alternative to traditional antibodies [23]. MIPs are synthetic recognition elements designed by incorporating specific template molecules during the polymerization process, resulting in binding sites that demonstrate high specificity and selectivity toward the target analyte [24]. At the end of a biochemical reaction, the resulting products are converted into measurable signals through a transducer. The type of transducer and the signal transmission mechanism used in a biosensor depends on the intended application and the specific biochemical process [25,26]. Various types of detector can be integrated into biosensor systems, among which optical and electrochemical detectors are the most commonly used due to their sensitivity and compatibility with diverse sensing platforms [27].

Even though optical sensor-based detection systems have become more common in recent years, electrochemical sensors are generally suitable for field applications owing to their lower cost and ease of integration into compact, simple devices. Compared to optical sensors, electrochemical sensors often avoid the need for complex arrangements with optical components and precise alignment, although modern optical set-ups can also be portable and user-friendly (e.g., fiber-based systems). They can be efficiently integrated with microelectrodes and electrochemical devices, making them well-suited for miniaturized analytical systems. Their simpler design also allows easier integration into portable and user-friendly platforms [11,12,13,28]. Unlike optical methods, electrochemical sensors are not affected by light absorption or scattering, facilitating accurate measurements even in turbid or opaque samples. Nevertheless, because electrochemical detection directly engages with the sample surface, it is more susceptible to fouling and non-specific adsorption than optical recognition [29,30]. Lab-on-a-chip (LoC) systems represents a significant advancement in the analytical sciences because this technology automates and simplifies complicated laboratory processes onto a single microfluidic chip. LOC platforms combine lots of procedures into a small device, including sample preparation, mixing, separation, and detection, allowing for quick and extremely accurate analyses and using very small volumes of sample and reagent [31]. Although their electrochemical integration enables rapid reactions, low detection limits (LODs), and quite a few analyses to identify pathogens, toxins, and other contaminants in complex food matrices, LOC platforms combine portability, speed, and cost-effectiveness with the increased sensitivity supported by nanomaterial integration in food safety applications [32,33,34]. Electrochemical devices in the food supply chain can be widely utilized to detect pathogenic bacteria such as *Salmonella*, *Listeria*, and *Escherichia coli* for evaluating freshness, nutritional value, and authenticity [35].

These systems are becoming next-generation platforms that guarantee food safety, regulatory compliance, and sustainability in contemporary agriculture and the global food supply chain by cutting down on analysis times, increasing sensitivity, and facilitating decentralized on-site testing. When electrochemical detection and microfluidics are combined, a new strategy with sensitive, portable, and reasonably priced devices can be developed to handle complicated matrices with accuracy and repeatability. In addition, LOC platforms efficiently control issues such as interferences and low analyte concentrations that are typical in field situations by combining sample preparation, separation, and detection into a single, compact structure [29,36,37,38]. The integration of smartphone technology into electrochemical devices can also guarantee improved portability, user-friendly interfaces, real-time data processing, cloud connectivity, and accessibility for non-specialists. Even in remote or resource-constrained environments, these connections turn smartphone-integrated electrochemical devices into mobile laboratories, providing quick, affordable, and easy-to-use biochemical studies. The efficiency and usability of point-of-care (POC) food and environmental tests are further enhanced by cellphones’ size, data processing capacity, and connection capabilities [39]. Therefore, smartphone-assisted electrochemical devices present promising ways to ensure food safety and sustainability in farming methods. However, to the best of our knowledge, no recent review has critically evaluated these instruments for monitoring contaminants in food and agriculture. Accordingly, this study reviewed the present advancements, working principles, applications, and future directions of such systems for the rapid, sensitive, and on-site detection of biological and chemical threats in agricultural and food systems.

## 2. Electrochemical Biosensors

Among biosensing platforms, electrochemical biosensors have emerged as an attractive choice thanks to their simplicity, low power needs, and compatibility with portable and POC devices [11,17]. This section reviews working principles, typical designs, and the role in detecting contaminants in agriculture and food. The low power requirement of electrochemical sensors represents a clear advantage, especially for use in areas with limited resources or infrastructure. With diverse enzymatic, immunological, and DNA-based sensing formats and wide applications in environmental monitoring, food safety, healthcare, and pharmaceuticals, electrochemical sensors have emerged as a more complex, cost-effective, and field-adaptable alternative to optical sensors [40,41]. In optical sensing, target detection commonly relies on nanomaterials like gold nanoparticles (AuNPs), graphene oxide (GO), or carbon dots, combined with fluorescent or chemiluminescent dyes to intensify signal and sensitivity. Beyond these conventional detection methods, advanced techniques are also employed in biosensing, including surface plasmon resonance (SPR) spectroscopy, optical waveguide light mode spectroscopy (OWLS), and piezoelectric quartz crystal microbalance (QCM) systems [42].

Nanomaterials such as GO and AuNPs significantly potentiate the sensitivity of electrochemical sensors by improving both signal production and analyte detection. AuNPs, owing to their high surface-to-volume ratio, provide a large number of active sites for immobilizing biomolecules such as enzymes, antibodies, or aptamers, thereby increasing capture efficiency. Their excellent electrical conductivity facilitates rapid electron transfer between the analyte and the electrode, whereas their inherent catalytic properties reinforce electrochemical processes, generating stronger signals at trace concentrations. In contrast, GO shows a substantial two-dimensional surface area decorated with oxygen-containing functional groups (–OH, –COOH, –O–), which support chemical functionalization and stable probe immobilization. Local pre-concentration and adsorption around the electrode interface are further enhanced by its π–π interactions with aromatic analytes. When GO is reduced to rGO (reduced graphene oxide), its conductivity is restored, which accelerates electron transmission and further enhances sensitivity. AuNPs and GO can produce hybrid platforms in which GO provides a high surface scaffold and AuNPs impart superior conductivity and catalytic activity, enabling detection of toxins, environmental pollutants, and food contaminants at pico- to femtomolar levels with notable sensitivity [3,11,17,38,40].

Moreover, electrodes modified with AuNPs or GO not only amplify the analyte signal but also maintain background noise at a limited level. As a result, nanomaterial-induced signal-to-noise ratio (SNR) enhancements improve sensitivity by lowering LODs and resulting in more reliable measurements [43,44]. In this regard, both biologically inspired amplification strategies, such as rolling circle amplification (RCA), and nanomaterial-based surface engineering present complementary approaches for strengthening the technical persuasiveness of next-generation DNA biosensors. For example, Gao et al. employed RCA as an alternative amplification method for DNA detection, generating long single-stranded DNA (ssDNA) products that meaningfully enhanced the electronic responses of silicon nanowire field-effect transistors (FETs). The abundant binding of repeated RCA sequences significantly increased the SNR, resulting in high sensitivity. When RCA was applied, the biosensor achieved an SNR > 20 at a detection level of 1 fM, demonstrating superior performance compared with most previously reported DNA sensors [45]. Similarly, nanomaterial-based surface modifications improve SNR by enlarging the effective electrode surface area and facilitating electron transfer, thereby further enhancing detection performance [44].

On the other hand, the integration of smartphones not only increases portability but also provides modern signal-processing capabilities. The central processing units (CPUs) and graphics processing units (GPUs) of advanced smartphones can efficiently execute computationally intensive algorithms, such as real-time noise filtering, Fourier transforms, and impedance spectrum analysis [46]. As a result, electrochemical signals, including voltammetric curves and impedance spectra, can be directly processed on the device with accuracy comparable to that of benchtop systems [47]. In addition, the coupling of these algorithms with wireless data transmission (Bluetooth, wireless fidelity (Wi-Fi), or cellular networks) provides real-time monitoring, rapid decision-making, and seamless integration with Internet of things (IoT)-based systems [48].

The detection methods used in electrochemical biosensors include amperometric, voltammetric, impedimetric, and potentiometric techniques. Each of these techniques has special advantages depending on the application. Potentiometric devices detect redox processes involving a bioreceptor, and the resulting biochemical interactions are transformed into qualitative and quantitative electrical signals [49,50]. This mode measures the potential difference between a working electrode and a reference electrode, typically in the presence of a counter electrode, without causing a sizable current to flow through the system. Such arrangements often use ion-selective electrodes (ISEs) as transducers, with membrane-based designs being the most prevalent [20,51]. The amperometric approach assesses the current produced during the redox reactions of electroactive materials by applying a constant voltage to the working electrode [52,53]. In amperometric sensors, the measured current is proportional to either the concentration of the electroactive species or the rate of the biocatalytic reaction. Conversely, voltammetry techniques such as differential pulse voltammetry (DPV), square wave voltammetry (SWV), and cyclic voltammetry (CV) apply a time-dependent voltage to the working electrode and monitor the current that results [54]. In voltammetry, the peak current observed within the linear potential window is proportional to the concentration of the electroactive species in solution, while different approaches, such as CV, SWV, DPV, and stripping voltammetry (SV), provide flexibility in experimental design. The characterization of electrode surfaces, pathogen detection, heavy metal ion monitoring, and other uses are made possible by impedimetric techniques, which use a low-amplitude voltage at different frequencies. Impedimetric methods are often preferred in biological systems over CV or DPV because they cause less disruption to the electroactive surface of the sensor, thus preserving its functional integrity. Due to these various electrochemical processes, biosensors are extremely versatile, reliable, and appropriate for field use in environmental monitoring and food safety [55,56].

Silver/silver chloride (Ag/AgCl) electrodes are frequently used as reference electrodes because of their reproducibility and durability. In potentiometric sensors, the measured potential is determined by the logarithm of the target ion activity in the solution. In addition to potentiometric methods, biosensing employs a range of electrochemical techniques, including conductometric, voltammetric, amperometric, and electrochemical impedance spectroscopy (EIS) [48]. Electrochemical techniques constitute the basis of sensing technologies in electrochemical devices because of their great sensitivity, quick reaction time, and affordability. Among them, voltammetric techniques (e.g., DPV and CV) are commonly used to examine redox-active materials. Nonetheless, amperometric and potentiometric approaches can be applied for highly reproducible real-time quantitative monitoring of target species. Developing the complex electrode materials, such as MXenes and carbon-based nanostructures, has profoundly increased surface area, charge transfer kinetics, and overall analytical performance [57]. These advancements are essential when the trace-level detection of contaminants is needed for agricultural and food safety monitoring. Recent research also emphasizes the function of flexible and hybrid composite electrodes, which increase the applicability of a range of portable sensing systems by providing superior electrochemical activity together with mechanical stability.

Electrochemical biosensor systems can be designed for both qualitative and quantitative analyses and have seen rapid growth in the global market, particularly in response to rising environmental and public health concerns after the COVID-19 pandemic. The market, valued at USD 16.9 billion in 2023, is projected to reach about USD 28.3 billion by 2032 [58]. The World Health Organization (WHO) highlights the development of diagnostic systems that meet the ASSURED criteria—an acronym for affordable, sensitive, specific, user-friendly, rapid and robust, equipment-free, and deliverable to end-users. These criteria promote the development of diagnostic tools that are cost-effective, accurate, accessible, and reliable [59]. Given the practical limitations of conventional analytical techniques, these requirements have driven growing interest in biosensor technologies [60]. Electrochemical techniques are particularly effective in the sensitive detection of water pollutants and toxic compounds. Even at very low concentrations, these substances can have serious biological effects. Detection of microbial and chemical food contaminants that threaten environmental safety, along with pollutants such as heavy metals, pesticides, pathogens, and infectious toxins that degrade soil quality and endanger water and air systems, remains a critical challenge. Addressing this challenge demands portable, highly sensitive technologies for on-site detection and monitoring [61]. Smartphone-integrated electrochemical devices are gaining significant attention thanks to recent advancements in flexible electronics and microfluidic technology. Biosensor mobility and field usefulness are enhanced by the development of lightweight, bendable, and wearable devices made possible by flexible electronics. They are especially well suited for food safety and agricultural monitoring applications due to their ergonomic designs, low power consumption, and mechanical stability [62]. However, microfluidics improves integration by allowing sample preparation, separation, and detection on a single chip. Microchannel design progress decreases analysis time and cost by providing an ultrasensitive and reproducible analysis of miniature sample amounts. These technologies, after their integration, open the door to the development of small, eco-friendly, and easy-to-use diagnostic tools that could revolutionize environmental monitoring, healthcare, and food safety [62,63].

## 3. Lab-on-a-Chip (LoC) Sensor System

**Figure 1:**
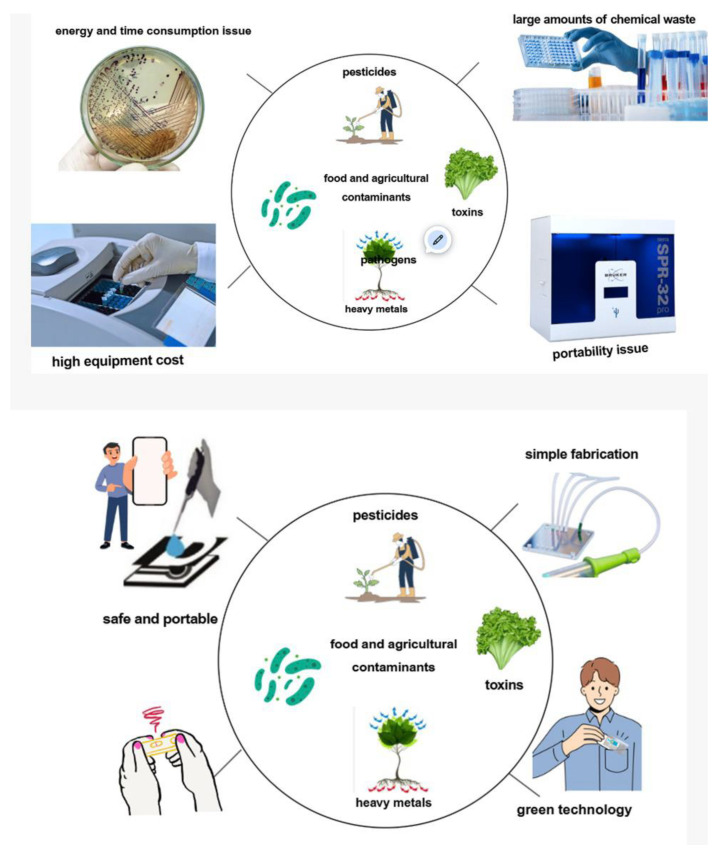
Advantages of LoC technology and comparison with traditional detection methods.

Delays in detecting critical food and agricultural contaminants, especially pathogens that can cause outbreaks, are often due to the need to send samples to remote laboratories. This process takes time because of transportation and lengthy analysis procedures. Although conventional detection methods, such as culturing, enzyme-linked immunosorbent assay (ELISA), and polymerase chain reaction (PCR), offer high accuracy [64], they are time-consuming, require skilled personnel, and are generally unsuitable for field applications. In recent years, portable and sensitive detection systems have been developed, however, many remain bulky, hard to transport, and dependent on external power sources or computers [65]. In contrast, handheld devices are compact systems weighing under 1 kg, operable with one hand, battery-powered, and equipped with integrated processors and displays [56]. LoC technology provides an excellent platform for such portable and real-time diagnostic systems. LoC devices use minimal sample volumes and integrate multiple laboratory processes on a single microchip. This technology can perform various functions, including sample preparation, mixing, dilution, electrophoresis, staining, and detection within a unified, compact system (Figure 1).

Recent market analyses indicate that the global market value of LoC devices was approximately USD 6 billion in 2023 and is projected to grow to about USD 6.29 billion in 2024. With an estimated compound annual growth rate (CAGR) of 7.47%, the market is expected to exceed USD 11.2 billion by 2032. These figures reflect the growing demand for compact, efficient, and field-deployable diagnostic technologies worldwide [66,67]. Recent studies report a rapid global increase in diagnostic tests, with approximately 209 million LoC-based COVID-19 tests conducted in the United States alone during the first half of 2021 [68,69]. The COVID-19 pandemic has accelerated the shift toward personalized medicine and highlighted the broader applicability of LoC technology—not only for detecting chronic diseases and influenza-like viral infections, but also for identifying foodborne contaminants such as pesticides, food pathogens, mycotoxins, and heavy metals [12,64,69,70].

Among the most significant advantages of LoC technologies are their high analytical sensitivity, ability to perform multiplexed analyses, user-friendly ergonomic design, and reduced biomedical waste. In diagnostic applications, the capacity of LoC systems to deliver faster and more accurate results enhances their clinical significance. The growing demand for compact diagnostic devices, increasing focus on biosensor and biomarker research, advances in manufacturing, and the search for cost-effective solutions have fueled interest in LoC-based systems. In this context, global investment in research and development related to LoC technologies has gained momentum. Recent developments demonstrate increasing investment and regulatory progress surrounding LoC technologies. For example, in November 2021, Parallel Fluidics secured USD 1.8 million in funding to scale the deployment of microfluidic LoC platforms. Similarly, in April 2021, PathogenDx received Emergency Use Authorization (EUA) from the U.S. Food and Drug Administration (FDA) for its DetectX-Rv test kit for COVID-19 diagnostics. Furthermore, in March 2022, Quotient Ltd. obtained CE certification from the European Union for its Extended Immunohematology (IH) microchip, integrated into the MosaiQ system. These examples illustrate the growing recognition and institutional support for innovative diagnostic technologies within the LoC domain [51,71].

The global rise in infectious diseases, coupled with advances in molecular biology and genetic engineering, has established the United States as a dominant player in LoC technologies. As of 2022, the U.S. held a leading market share of 45.8%. In Europe, Germany has the largest market, while the United Kingdom is the fastest growing. In the Asia-Pacific region, rapid adoption of healthcare technologies, the rising prevalence of chronic illnesses, and structural reforms in health systems have all driven the region’s growing potential. Within this context, China holds the largest market share in the region, whereas India leads in growth rate. Although microfluidics-based platforms had already established a strong presence in both industrial and academic contexts by 2022, POC electrochemical devices are also becoming increasingly prominent in clinical applications. POC systems that integrate with mobile devices, particularly smartphones, enable diagnostic procedures to be performed outside conventional hospital settings. Given this capability, POC diagnostics are expected to become one of the fastest-growing subsegments of the LoC market in the near future [64,65,66,67,69,71,72].

Regional disparities in agricultural practices necessitate adaptive sensor design strategies. In smallholder or resource-limited settings, affordability, robustness, and independence from external power sources are critical, with paper-based or capillary-driven μPADs offering portable, low-cost alternatives [73]. Conversely, industrial farming systems can support more advanced IoT-enabled LoC devices with multiplexing capabilities and cloud connectivity, enabling high-throughput, real-time monitoring [74]. Communication technologies must also adapt to context, with Wi-Fi typically deployed in urban or industrial farms, while long range wide area network (LoRaWAN) or other low-power wide-area networks are better suited for rural and remote environments [75]. Importantly, scalability remains a major challenge for low-income farmers, where economic and infrastructural barriers constrain adoption [76]. Addressing these disparities through tailored sensor platforms will contribute to global applicability and equitable food safety monitoring.

**Figure 2:**
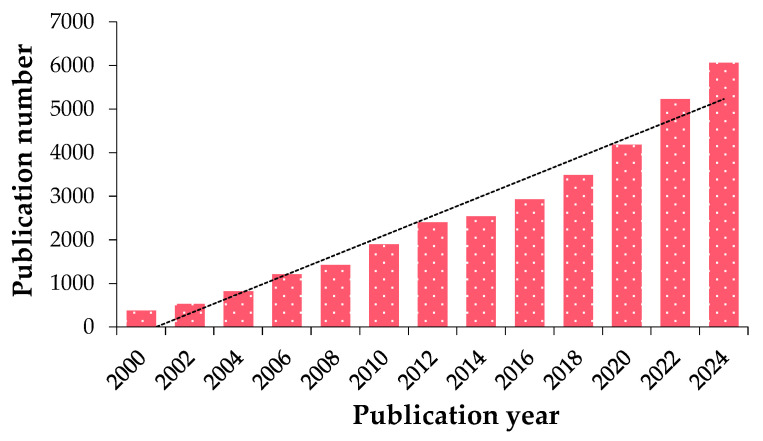
Publications with the topics “LOC” from 2000 to 2025. Numbers were collected from the WOS platform by searching the aforementioned topics and refining the publication years from 2000 to 2025.

Using the keywords “lab-on-a-chip” and “electrochemical biosensor,” an examination of the Web of Science (WOS) database reveals a notable upward trend in scholarly articles on LoC technology from the early 2000s, with a particularly notable increase in the last five years. Between 2000 and 2025, the number of research outputs in this field has grown approximately sixteen-folds. This growth is illustrated in the dendrogram in Figure 2 [77].

### 3.1. LoC Platforms: Design and Development

LoC platforms, also known as micro total analysis systems (μTAS), are compact analytical devices that integrate multiple laboratory processes onto a single chip, typically ranging in size from a few millimeters to several square centimeters [78,79]. In designing these systems, particular emphasis is placed on mechanical flexibility, electrical conductivity, and biocompatibility [80]. The fabrication of LoC devices involves various techniques such as photolithography, screen printing, injection molding, and electroplating. A wide array of materials, including ceramics, glass, silicon, photoresists, acrylates, thermosets, and polydimethylsiloxane (PDMS), is chosen based on the desired properties and application [81]. Silicon has long been a preferred material because of its semiconducting nature and the presence of silanol groups that enable effective surface modification. However, its opacity to visible light and relatively high production cost limit its use in certain optical applications [82,83]. Nevertheless, glass presents excellent optical transparency, chemical stability, and biocompatibility, making it ideal for high-performance analytical tasks. Despite these advantages, its expensive fabrication and the use of hazardous chemicals like hydrofluoric acid remain significant drawbacks. Among LoC materials, PDMS is the most commonly used elastomer due to its ease of processing, flexibility, chemical inertness, gas permeability, and strong adhesion to various substrates, making it particularly well suited for constructing microfluidic devices [84].

**Figure 3:**
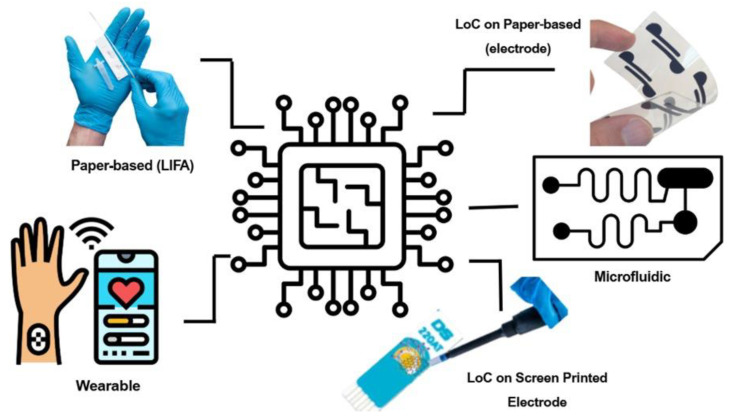
Different types of LoC biosensors and their technology.

Among the most frequently used microfabrication techniques for producing LoC platforms are photolithography, soft lithography, 3D printing, laser micromachining, and screen printing. Photolithography presents high-resolution patterning, achieving features as small as 5 μm, and is commonly used in microfluidic device fabrication [60]. However, its widespread use is limited by high operational costs, complex equipment, and the need for chemically pure environments. In contrast, soft lithography is a cost-effective, micro- and nano-scale patterning technique that can produce structures ranging from 30 nm to 100 µm. Despite its versatility, this method has limitations in resolution, mechanical robustness, scalability, and alignment precision, which restrict its application in large-scale or high-accuracy manufacturing [85]. Nevertheless, thanks to its accessibility and flexibility, soft lithography remains a basic technique in microfabrication processes. Laser micromachining has been successfully applied in the mass production of thermoplastic molds, reducing production time by up to 90% and achieving surface roughness as low as ~65 nm [85]. This technology enables rapid prototyping without the need for cleanroom facilities, supports a wide range of materials, and can be integrated with advanced measurement systems such as in situ light detection and ranging (LiDAR) profiling [86]. Thus, laser micromachining has become a prominent method for fabricating microfluidic components. 3D printing technologies have gained attention for LoC applications because of their ability to produce rapid prototypes and high-resolution microscale structures without cleanroom conditions. By using specially formulated low-viscosity inks, it is possible to fabricate open and embedded microfluidic structures with precise horizontal and vertical resolutions [87]. Inkjet printing provides the advantage of maskless, fast production and high resolution [88]. Screen printing is another widely used technique in the development of electrochemical devices, owing to its low cost, scalability, and ease of application on various substrates such as polyethylene terephthalate (PET), paper, PDMS, and glass [89]. Using conductive inks, enzymatic reagents, or nanomaterials, this method allows the formation of electrode arrays, microchannels, and reaction zones. Screen printing is particularly well-suited for electrochemical detection platforms and has been successfully employed in detecting glucose, pesticides, heavy metals, mycotoxins, and pathogens [90]. Moreover, integrating screen-printed sensors with smartphone-compatible systems and paper-based microfluidic platforms has made this approach practical in resource-limited settings [91]. For instance, screen-printed sensors developed to detect OP pesticide residues in vegetables have demonstrated LODs below regulatory thresholds, delivering reliable results in enzyme-based electrochemical tests [92]. In addition, LoC platforms incorporating printed electrodes and lateral flow systems have been used for the rapid, on-site identification of environmental contaminants. Figure 3. Schematic representation of different types of electrochemical biosensors, including paper-based, screen-printed electrode-based, wearable, and microfluidic platforms, indicating their integration into nano-biosensing technologies. Despite certain limitations in resolution, screen printing remains a practical and effective technique for commercial electrochemical device manufacturing due to its simple production process, compatibility with biomaterial-based inks, reproducibility, and affordability.

### 3.2. Cutting-Edge LOC Technologies for Food and Agricultural Contaminant Detection

Interest in LoC technologies has increasingly grown in recent years. They provide portable, fast, and highly sensitive tools for detecting contamination in food and agriculture. According to WOS data, 47 research articles and reviews on LoC technologies for food and agricultural pollutant detection had been published by 2025.

Zou et al. [93] developed a miniature LoC device using 3D printing for rapid on-site detection of thiamethoxam pesticide. The device integrates immunochromatographic analysis and sample pre-treatment into a single platform. It was designed to filter and purify raw food samples before analysis. Tested on 13 different food matrices, the system showed high sensitivity with an IC_50_ value of 0.10 ng mL^−1^. Each analysis was completed in less than 15 min. Accuracy was confirmed against liquid chromatography–tandem mass spectrometry (LC-MS/MS), showing recovery rates of 87.2–107.4% and relative standard deviations (RSDs) of 2.1–7.6%. This handheld system is among the first fully integrated LoC platforms that can perform both sample preparation and pesticide residue analysis directly in the field. It is portable, reliable, and easy to use, making it a promising tool for real-time food safety monitoring [93].

Atoloye et al. [94] developed a LoC platform called Rhizosphere on a Chip (Rhizo Chip) to study root exudation in industrial hemp (*Cannabis sativa* L.). The system incorporates soil-like minerals, such as potassium feldspar, biotite, and kaolinite, into the microfluidic environment, creating growth conditions similar to natural soils. These minerals greatly improved root development in hemp seedlings, indicating their vital role in root physiology. Untargeted metabolomic analysis identified 170 compounds, including organic acids, amino acids, and secondary metabolites, with clear variation based on the mineral composition of the substrate. This technology allows long-term, high-resolution monitoring of root exudates in non-model crops and supports valuable insights for sustainable agriculture [94].

**Figure 4:**
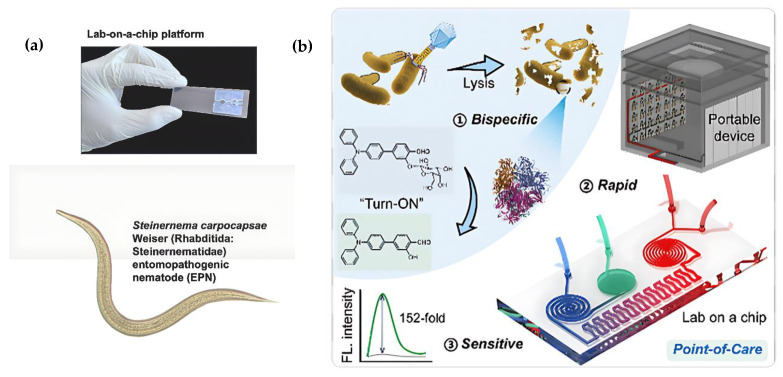
(**a**) LOC system for studying behavioral responses of the entomopathogenic nematode *Steinernema carpocapsae* to host-derived chemical stimuli (reprinted from Manduca et al. [95]), and (**b**) an innovative LoC platform combining AIEgen-based signal reporters with microfluidic technology for the rapid detection of *E. coli* O157:H7 (reprinted with permission from Feng et al. [96]. Copyright © [2025] American Chemical Society).

The structural designs of some LoC platforms, such as those reported by Manduca et al. [95] and Feng et al. [96], are highly flexible. They show strong potential for integration with electrochemical measurement systems for future multifunctional sensing applications, even though their original design focused on optical detection (Figure 4).

In a related study, Wang et al. [97] developed a next-generation analytical system for rapid field detection of veterinary drug residues in food. This system combines a portable magnetic relaxation switch (MRS)-based biosensor with a compact nuclear magnetic resonance (NMR) device. The compact system integrates sample preparation, analyte identification, and quantitative analysis on a single platform. It shares many characteristics of LoC systems, presenting a practical alternative to conventional laboratory methods. The device detected norfloxacin in under 15 min, with a detection range of 5–100 ng mL^−1^ and an LOD of 1.63 ng mL^−1^. A strong linear correlation (R^2^ = 0.98) confirmed its accuracy. This micro-NMR-based system is dependable, portable, and sensitive, revealing the real-world potential of miniaturized analytical devices for food safety [97]. In summary, LoC technologies are rapidly evolving into versatile, field-ready platforms with great potential for food safety and agricultural monitoring.

## 4. Smartphone-Integrated Electrochemical LoC Systems

### 4.1. Smartphone-Based Biosensors with LoC Technology

Smartphones have become widely accessible and prevalent worldwide, with approximately 54% of the global population using one. This widespread adoption positions smartphones as a powerful tool for decentralized solutions, particularly in healthcare and diagnostics, acting as an alternative to centralized laboratory systems [98]. Compared to microcontroller units (MCUs), such as Arduino, and single board computers (SBCs), such as Raspberry Pi, smartphones provide a more compact, user-friendly, and fully integrated platform. Although MCUs and SBCs are low-cost and highly customizable, they lack the built-in sensors, advanced hardware, and seamless software environment that smartphones provide. Hence, smartphones have become the preferred choice for mobile health applications and portable diagnostic systems [99,100]. Therefore, custom analytical devices based on MCUs or SBCs are typically larger in size, lower in performance, dependent on external components, and more difficult to operate. In contrast, smartphones are more robust, ergonomically designed, and offer superior sensor integration, making them an ideal choice for field-based analytical applications [73,101]. Moreover, smartphones integrate perfectly into digital data infrastructures, providing a powerful platform for machine learning (ML) and artificial intelligence (AI)-driven applications in health, agriculture, and food safety. Through wireless technologies such as Bluetooth and Near Field Communication (NFC), smartphones enable both power delivery and data transmission without physical connections. This capability supports the development of portable, low-cost, battery-free biosensor platforms [102,103]. Smartphones equipped with NFC modules are increasingly used for biological fluid analysis, biochemical detection, and real-time food safety monitoring, highlighting their versatility and growing role in decentralized diagnostics and environmental monitoring [104]. Microfabrication of microfluidic channels (often using soft lithography or photolithography), deposition of conductive materials to form electrodes (e.g., carbon, gold, or graphene-based screen-printed electrodes), surface functionalization with recognition elements (e.g., enzymes, aptamers, or antibodies), and integration with microcontrollers or smartphone interfaces are regular manufacturing steps for electrochemical devices integrated with smartphones [105]. Calibration and validation against standard laboratory equipment are usually the next steps, after which the device is packaged and sealed to guarantee consistency, portability, and sterility. Attaining reproducibility and scalability as two prerequisites for commercialization requires these actions [32].

#### 4.1.1. Integration of Smartphones into Electrochemical Sensing Systems

**Figure 5:**
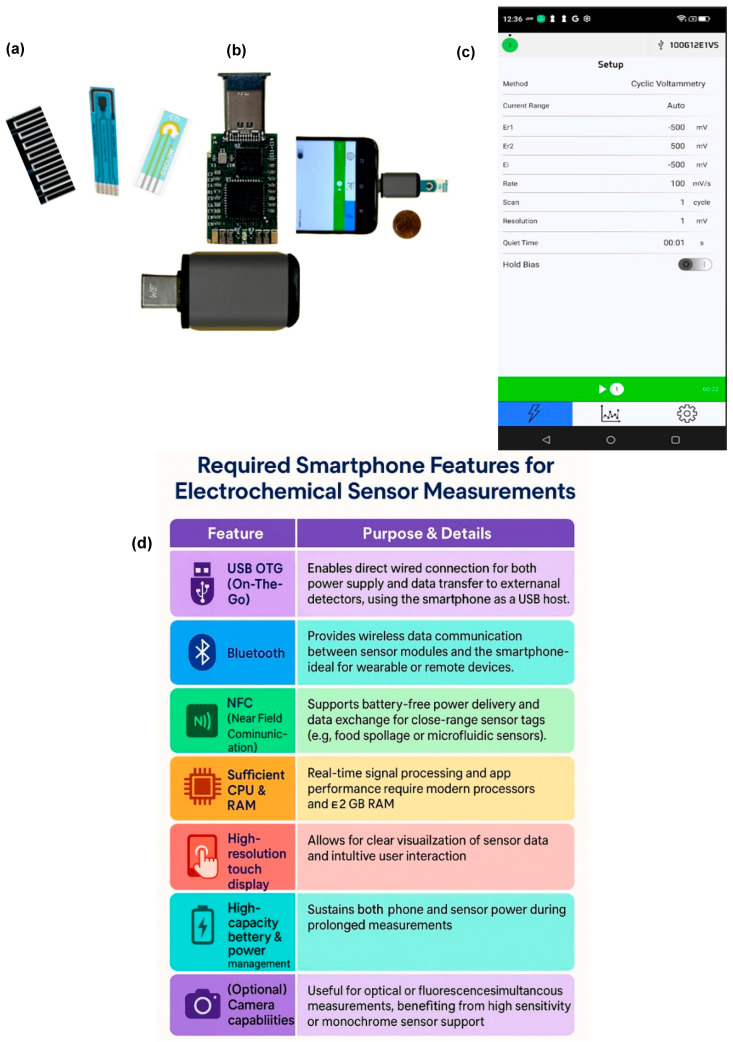
Components of the smartphone electrochemical measurement system. (**a**) Different kinds of electrode, (**b**) detectors of the system, and (**c**) smartphone equipped with measurement applications (reprinted from Savas et al. [16]), and (**d**) required smartphone features for electrochemical sensor measurements.

The widespread adoption, portability, and modern features of smartphones have driven their increasing integration into electrochemical detection technologies. Figure 5 illustrates the components of a smartphone-based electrochemical measurement system, including various electrode types, detectors, and a smartphone equipped with measurement applications [16]. By combining embedded electrochemical sensors, mobile software, wireless connectivity, cloud-based storage, and ML, smartphones are reshaping diagnostics in healthcare, food safety, and environmental monitoring [102,106]. This change is reshaping point-of-need testing by making it more efficient, accurate, and accessible. It allows reliable diagnostics outside conventional laboratories. Smartphones improve the efficiency of electrochemical sensing platforms by combining powerful hardware (processors, cameras, and NFC modules) with advanced software. Real-time data collection, wireless transfer, and cloud analysis facilitate the fast detection of viruses, pesticides, and heavy metals, even in complex food samples. Mobile applications improve usability by allowing non-specialists to run tests, view results instantly, and share data digitally [73,98,99,100,101,102,103,106]. Compared to traditional portable readers, smartphones reduce size, cost, and complexity, making them ideal for decentralized food safety and agricultural monitoring [107].

#### 4.1.2. Smartphone-Based Electrochemical Device Architecture and Workflow

Smartphone-based electrochemical sensing systems are gaining prominence due to their portability, user-friendliness, and integration capabilities. A standard platform typically consists of three main components: an electrode system, a microcontroller unit or miniature potentiostat, and a smartphone interface. The electrochemical detector, or potentiostat, serves as the central unit, receives commands from the smartphone, performs measurements, and sends data back for real-time analysis. The device architecture generally includes a power module (with a lithium-ion battery and voltage regulators), a microcontroller unit (e.g., STM32F1), a digital-to-analog converter (DAC), a noise filtering circuit, a constant potential circuit to maintain stable voltage across electrodes, a Bluetooth communication module, and a signal detection module that enhances sensitivity [108]. The process begins when the smartphone connects to the detector via Bluetooth. The user inputs measurement parameters through a mobile app, which the microcontroller interprets to regulate the DAC and generate the appropriate voltage signal. This signal passes through pre-bias and filtering circuits before reaching the electrodes, where it triggers an electrochemical reaction. The resulting current is then digitized and sent back to the smartphone for display and analysis [109,110]. To validate system performance, a screen-printed three-electrode configuration, comprising a carbon working electrode, an Ag/AgCl reference electrode, and a carbon counter electrode, was used. Tests were carried out in standard redox solutions using various electrochemical modes such as chronoamperometry (CA), CV, linear sweep voltammetry (LSV), and DPV. Compared with a commercial electrochemical analyzer, the system showed less than 0.5% deviation in CV mode, indicating high accuracy and reliability [111,112]. However, there are some significant obstacles to commercializing smartphone-integrated electrochemical devices. Consistent and repeatable sensor production can guarantee the long-term storage stability of biorecognition elements and handle signal interference in complex food or environmental samples. Economic and industrial hurdles include achieving regulatory approval criteria for food safety and agriculture monitoring, harmonizing processes across various devices, and increasing production while maintaining low costs. Large-scale adoption is further hindered by problems such as reliance on throwaway plastics, short battery life for field applications, and the requirement for interdisciplinary user training. Translating present research into generally accessible, commercially ready products will require overcoming these obstacles [113]. In conclusion, these smartphone-integrated electrochemical systems provide an innovative and practical solution for decentralized diagnostics, with potential applications in clinical, agricultural, and environmental domains thanks to their compact design, real-time data processing, and compatibility with advanced data analysis tools.

## 5. Trends in Lab-Chip Electrochemical Biosensors for Food and Agricultural Contaminants

**Figure 6:**
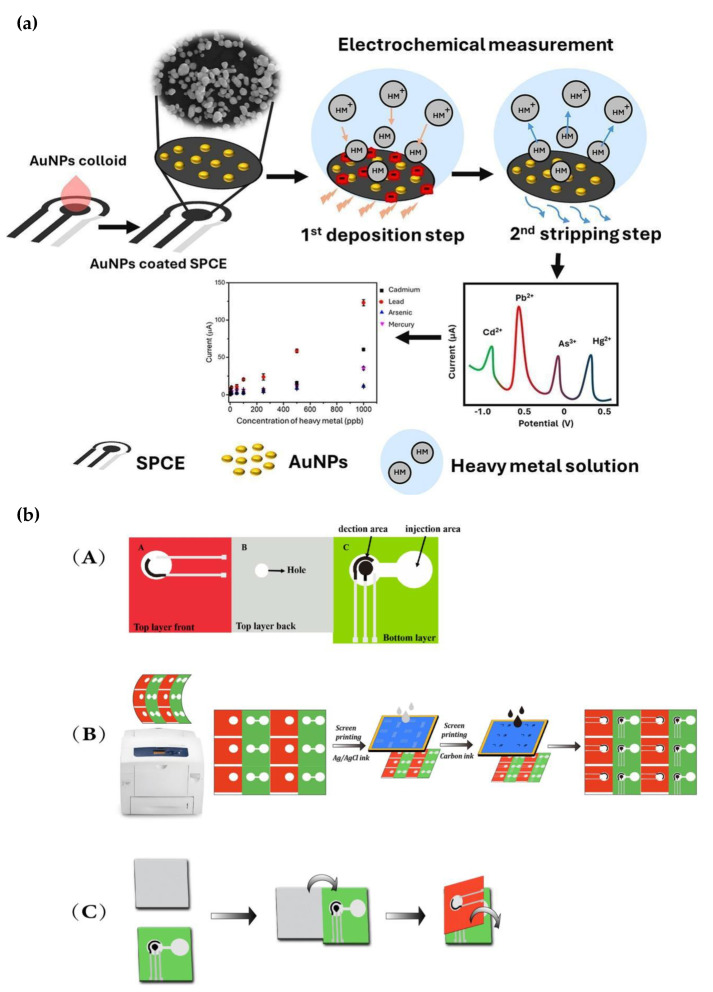
(**a**) SPCE modified with AuNPs, enabling the concurrent detection of Cd^2+^, Pb^2+^, As^3+^, and Hg^2+^ ions in the water (reprinted with permission from Karn-Orachai et al. [120]. Copyright © [2025] Elsevier), and (**b**) Design (A), fabrication (B), and assembly (C) of the EµPAD for detecting Cd, Pb, and Hg (reprinted with permission from Wei et al. [121]. Copyright © [2025] Elsevier).

In recent years, scientists have placed a high premium on the sensitive, precise, and reliable detection of heavy metal ions in environmental samples due to the growing pollution and its detrimental effects on human health. Specifically, the development of portable, easily navigable electrochemical devices with low LODs has enabled practical solutions for both laboratory and field conditions. These advancements have accelerated environmental monitoring by providing on-site and real-time analysis of heavy metal pollution [114,115,116]. Khoobi et al. used an ultrasonic process to develop zinc hexaferrite nanostructures using starch as a reducing agent. When electrodes were modified using the resultant nanostructures, they demonstrated high sensitivity for detecting mercury ions (Hg^2+^). Using the DPV approach, the sensor attained an ultra-low LOD of 0.20 nM and functioned over a broad linear range of 0.004–900.0 µM [117]. Similarly, Dongyang et al. modified graphite powder-based electrodes with nine distinct inexpensive nanoporous organic polymers (NOPs) to create a novel device for the trace-level detection of heavy metals. These composite electrodes demonstrated great sensitivity and repeatability with a very low LOD of 0.0003 μg L^−1^ and an RSD below 7.4%. A major addition to the area is indicated by this study, which is the first to report the application of NOPs in electrochemical sensing systems [118]. Liu et al. carried out another investigation into the biological detection of heavy metals. They created a bio-electroactive sensor by coupling a GO hydrogel made by bio-reduction with yeast cells that have glucose oxidase enzymes on their surface. With recovery rates ranging from 88% to 106.5% in actual wastewater samples, this sensor showed promise and attained an LOD of 17.0 µM for Cu^2+^ ions [119]. Karn-Orachai et al. focused on the simultaneous detection of heavy metals and created a sensor based on screen-printed carbon electrodes (SPCE) modified with AuNPs. This sensor helped detect Cd^2+^, Pb^2+^, As^3+^, and Hg^2+^ ions simultaneously. Using DPV, the sensor produced low LODs of 1.5 ppb for Hg^2+^, 4.4 ppb for Pb^2+^, 7.6 ppb for As^3+^, and 9.4 ppb for Cd^2+^, proving high accuracy and stability in environmental water samples [120] (Figure 6a).

Another portable approach was proposed by Wei et al., who developed an origami-style electrochemical microfluidic paper-based analytical device (EµPAD). Figure 6b illustrates the paper-based screen-printed electrode prepared using manual brushing with various commercial inks. Modified with nitrogen-doped graphene (NG), this sensor provided a linear response in the range of 5–100 µg L^−1^ using the LSV technique. It achieved LODs of 0.5698 µg L^−1^ for Cd^2+^, 0.4024 µg L^−1^ for Pb^2+^, and 0.2565 µg L^−1^ for Hg^2+^. Moreover, the sensor demonstrated high reliability with RSD below 7% and recovery rates ranging from 96.4% to 106.2% [121]. Finally, Han et al. could real-time detect Pb^2+^ and Hg^2+^ ions in tap and agricultural water using a compact electrochemical sensor network integrated with a WeChat mini-application. This system illustrates how mobile technologies can improve environmental monitoring [122]. Overall, studies conducted in 2025 highlight that portable electrochemical sensors developed for direct, on-site detection of heavy metal ions in environmental samples, especially when combined with LoC technologies, show high sensitivity, selectivity, and ease of use.

Pesticides are one of the most important contaminants of agricultural and food products. They have played a major role in modern agriculture, substantially improving crop yields in recent decades [122,123,124]. However, their widespread use has raised serious concerns about environmental sustainability and human health. Pesticide residues, often accompanied by heavy metals such as cadmium and mercury, can accumulate in staple foods like rice, posing risks to the nervous system, development, and vital organs. In response to growing food safety concerns, the global pesticide residue detection services market was valued at USD 1.23 billion in 2025 and is projected to grow at a CAGR of 5.3% through 2033 [123]. This growth is largely driven by stricter regulatory policies and rising consumer demand for pesticide-free products. Although chemical detection methods remain widely used, biological and biosensor-based technologies are gaining popularity due to their high sensitivity and portability [73]. Leading companies such as Eurofins and SGS are investing heavily in research and development to enhance detection capabilities and meet emerging global standards. In the European Union, strategic frameworks like the Farm-to-Fork initiative aim to reduce pesticide risks. However, current monitoring systems still fall short of accurately assessing the exposure of people living near agricultural areas [123,124,125,126]. To address these shortcomings, France has developed new risk indicators based on spatial pesticide application data, and the upcoming Statistics on Agricultural Input and Output (SAIO) regulation—set to take effect in 2026—will require professional users to digitally record pesticide use, improving data accuracy and accessibility. Ultimately, advancing detection technologies and promoting data quality and sustainable practices such as integrated pest management are essential for balancing agricultural productivity with protecting public health and the environment [122,123,127,128].

**Figure 7:**
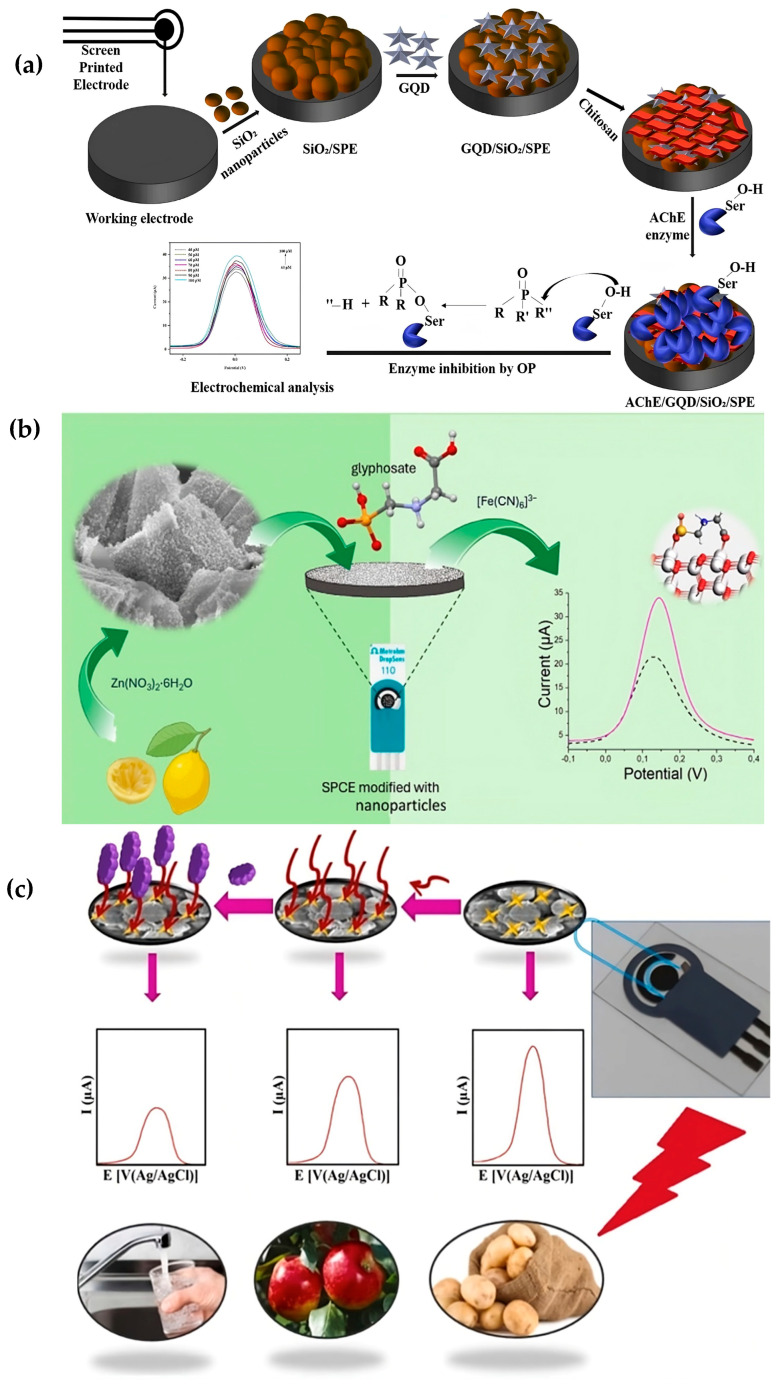
(**a**) Electrochemical biosensor for malathion detection was developed using AChE inhibition (Reprinted with permission from Yadav et al. [130]. Copyright © [2025] Elsevier), (**b**) A disposable, eco-friendly electrochemical sensor platform enabling field detection of glyphosate (Reprinted from Novakovic et al. [128]), and (**c**) The method of fabricating of CBA from food and water (Reprinted from Khosropour et al. [133]).

Wen et al. have recently designed an electrochemical, dual-mode LoC platform using a COF/methylene blue@MnO_2_ (COF/MB@MnO_2_) composite for detecting organophosphorus pesticides (OPs). This system combines electrochemical and photothermal measurements, enabling highly selective, sensitive, and cost-effective detection of OPs. The sensor achieved an LOD of 0.0632 ng mL^−1^ in electrochemical mode, indicating strong potential for field applications. With an LOD of 0.0632 ng mL^−1^ in electrochemical mode, the sensor showed great promise for field use [129]. Similarly, to detect malathion, a common and dangerous OP, Yadav et al. created an electrochemical biosensor based on acetylcholinesterase (AChE) inhibition. AChE was immobilized onto a screen-printed electrode that had been altered using graphene quantum dots (GQDs) and silica nanoparticles (SiO_2_) to create the biosensor. The sensor demonstrated a sensitivity of 0.10 µA pM^−1^, a working range of 40–100 pM, and an LOD of 0.01 ppm when using SWV (Figure 7a). Its suitability for field-based monitoring was confirmed by its successful detection of malathion in several vegetable samples [130]. A fenitrothion (FNT)-specific electrochemical sensor based on porous GO (PGO)-modified SPCE was created by Balasubramanian et al. to detect FNT, a herbicide with a high environmental toxicity. The performance of the sensor was assessed using SWV, CV, and EIS. It displayed a broad linear range of 0.02–250 µM and an LOD of 0.061 µM. The sensor effectively detected FNT residues in fruit and water samples and revealed potential for integration into portable, field-deployable devices [131,132].

A single-use, environmentally friendly electrochemical device was developed by Novakovic et al. to detect glyphosate, which makes up approximately 72% of all pesticide use worldwide. Under lab circumstances, the sensor’s linear range was 0.5 µM to 7.5 mM, its LOD was 0.648 µM, and its detection time was 30 min. The LOD in actual river water samples was 0.96 µM, indicating that it is appropriate for environmental monitoring (Figure 7b) [103]. Khosropour et al. introduced a novel nanocomposite-based electrochemical aptasensor for detecting carbaryl (CBA), a broad-spectrum carbamate insecticide known for its adverse health effects. Using DPV, the sensor showed a linear range of 1.0 pmol L^−1^ to 0.1 µmol L^−1^ and an LOD of 0.4 pmol L^−1^. It demonstrated high selectivity, repeatability (RSD = 2.96%), reproducibility (RSD = 3.14%), and stability (92%) in real samples such as tap water, apple juice, and potato juice (Figure 7c) [133]. To sum up, these studies show the growing importance of electrochemical sensor technologies in the rapid and accurate detection of pesticide residues and heavy metals. The integration of electrochemical devices with LoC systems with IoT technologies presents a promising pathway for real-time monitoring and regulation of pesticide use directly in the field.

Microplastics, veterinary drug residues, per- and polyfluoroalkyl substances (PFAS), pesticide metabolites, and other emerging contaminants introduce substantial hazards to ecosystems, food safety, and human/animal health. Their potential for bioaccumulation, toxic effects, and widespread distribution make their early and on-site detection critically important [134]. Although conventional chromatographic techniques have high accuracy, they are limited by the need for laboratory infrastructure, long analysis times, and high costs, making them inadequate for rapid decision-making in field applications [3]. In this regard, smartphone-integrated LoC electrochemical devices are as powerful alternatives, presenting advantages such as low cost, portability, short analysis time, and real-time data transfer.

Smartphone-assisted LoC electrochemical platforms have recently received considerable attention, mainly for the rapid and sensitive detection of microplastics. Xiao et al. [135] developed a single-particle electrochemistry-based system on carbon-coated microwires, where collisions of microplastics with the electrode generated oxygen reduction spikes that correlated with particle concentration. They integrated a cadmium sulfide/cerium dioxide (CdS/CeO_2_) heterojunction-based photoelectrochemical–electrochemical dual-mode sensor with a smartphone-controlled portable potentiostat, resulting in ultralow LODs for polystyrene nanoplastics (as low as 0.38 ng/mL). Also, a self-powered dual-photoelectrode system employing a bismuth oxysulfide (Bi_2_O_2_S) photoanode and a copper bismuth oxide (CuBi_2_O_4_) photocathode operated without external energy input and provided reliable performance in real wastewater samples with an LOD of 0.09 μg/mL and high selectivity [135].

Other innovative approaches include EIS-based “electronic tongue” concepts enabling ML-assisted classification of PET microplastics of different sizes [136], biomimetic electrode surfaces replacing fluorescent peptide modifications in EIS platforms [137], CeO_2_ nanoparticle-modified glassy carbon electrodes for sensitive detection of polyethylene and polypropylene residues in seawater [138], and chitosan–magnesium oxide (MgO) nanocomposite electrodes for electrochemical determination of the plastic additive hexamethylenetetramine [139]. Furthermore, Lee et al. [140] employed single-entity electrochemistry to visualize polystyrene and polypropylene microplastics in real time at ultramicroelectrodes. Du et al. [141] quantified polystyrene microplastics over the range of 0.01–25 mg/L using graphene-based EIS electrodes derived from petroleum waste. Gongi et al. achieved ultrasensitive detection of various microplastics (10^−11^ M) using cyanobacterial extracellular polymers [142], whereas Colson and Michel demonstrated continuous monitoring of 212–1000 μm microplastics using a multichannel EIS flow-cell platform with high recovery rates [143].

LoC-based electrochemical platforms combined with smartphone interfaces have also been applied to detect veterinary drug residues. Dogra et al. developed a graphene oxide/multi-walled carbon nanotube nanocomposite electrode for chloramphenicol, achieving a detection limit of 46 nM and a linear range of 0–600 μM. The system was compatible with both benchtop Autolab and pocket-sized Palmsens potentiostats and validated in milk, serum, and water samples with high recoveries [144]. Furthermore, aptamer-based LoC electrodes have been developed for drugs such as meloxicam, enrofloxacin, and florfenicol, where the specific binding affinity of aptamers provided high selectivity and reusability [61].

Beyond microplastics and veterinary drugs, smartphone-assisted LoC electrochemical devices are also being developed for the detection of PFAS, pharmaceuticals and personal care products/endocrine-disrupting chemicals (PPCPs/EDCs) (e.g., bisphenol A (BPA)), mycotoxins, algal toxins, pesticide residues/metabolites, and engineered nanomaterials. For PFAS, metal–organic framework (MOF)-integrated impedimetric microfluidic chips demonstrated ppt-level detection of PFOS through concentration-dependent impedance changes [145,146], while wearable/portable electrochemical devices have also shown feasibility for field applications. For PPCPs/EDCs, aptamer/nanoparticle-modified voltammetric sensors on SPCEs achieved μM–nM sensitivity for BPA, and these systems are typically paired with Bluetooth-enabled handheld potentiostats and smartphone apps for real-time reporting [147,148].

In the case of mycotoxins and algal toxins, LoC-based EIS and voltammetry aptasensors are increasingly common. For example, microchannel “comb-shaped” aptasensors for aflatoxin B1 (AFB1) enabled rapid screening in grains, whereas a hexaammine-ruthenium(III) ([Ru(NH_3_)_6_]^3+^) displacement-based aptasensor for microcystin-LR (leucine (L) and arginine (R)) achieved an LOD of 9.2 pM with response times under 15 min, adapted to smartphone-controlled portable analyzers [149,150]. Pesticide residues and metabolites have been addressed with MIP-based smartphone-integrated electrochemical chips, such as Au/rGO/SPCE-MIP electrodes for thiamethoxam, providing quantitative results with portable readers [151,152,153].

**Figure 8:**
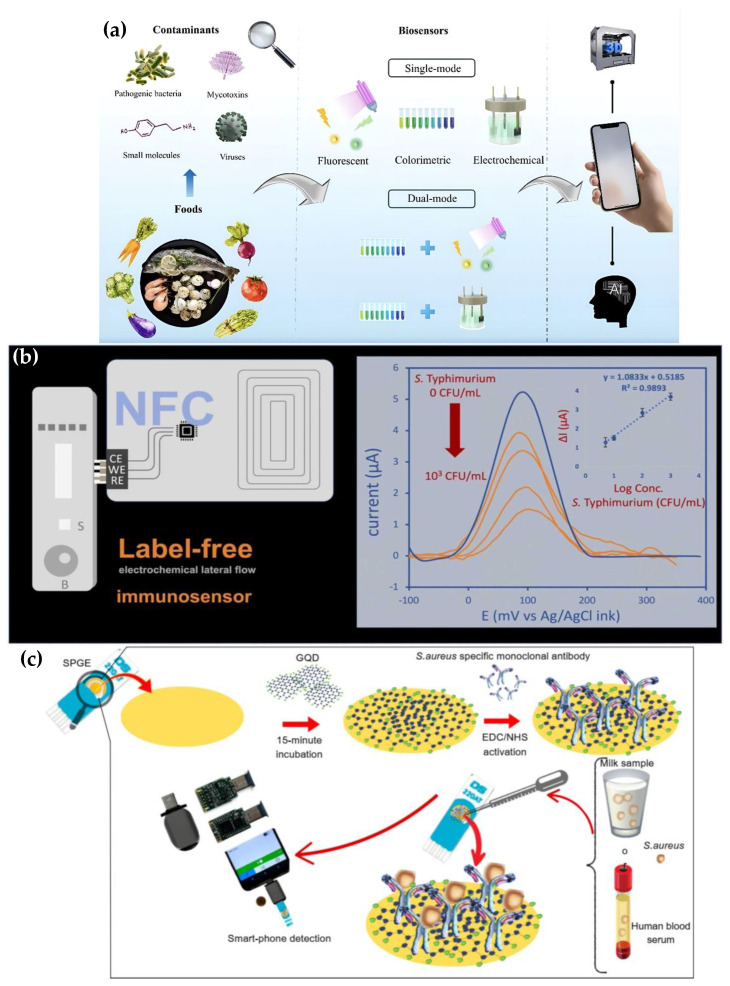
(**a**) The principles of a smartphone-based detection system for food contaminants (reprinted with permission from Qin et al. [154]. Copyright © [2025] Elsevier), (**b**) label-free electrochemical lateral flow immunosensor integrated with NFC technology for detection of *S. typhimurium* in food samples (reprinted with permission from Yentongchai et al. [155]. Copyright © [2025] Elsevier), and (**c**) schematic illustration of the smartphone-based label-free GQD immunosensor for *St. aureus* detection in undiluted milk and human blood serum (reprinted from Savas et al. [16]).

Foodborne pathogens remain among the top three contaminants posing serious risks to food safety. Early detection is critical to preventing outbreaks from contaminated food [154,155,156,157,158]. In recent years, innovative biosensor technologies have facilitated the development of portable, field-deployable diagnostic systems. These technologies provide not only rapid pathogen detection but also help identify contaminated food sources directly at the point of need, contributing to faster response and more effective outbreak control. Among these, smartphone-integrated electrochemical biosensors, particularly LoC platforms and other electrochemical devices, have emerged as promising tools for rapid and on-site detection of foodborne pathogens. By combining disposable microfluidic patches with wireless modules, they enable fast, ‘sample-in-result-out’ testing with minimal sample volumes, suggesting a portable and scalable solution for POC food safety monitoring [120,133,154,155,156,157,158]. Figure 8a illustrates how biosensors detect various food contaminants, such as pathogenic bacteria, mycotoxins, small molecules, and viruses, in food products. It shows single-mode biosensors (fluorescent, colorimetric, electrochemical) and dual-mode biosensors that combine these techniques for improved detection. The integration with smartphones, AI, and 3D-printed components further enhances the portability, data analysis, and usability of these diagnostic tools [154].

Govedarica et al. [156] made highly conductive gold leaf electrodes (GLEs) by laminating 24-karat gold leaves onto polyvinyl chloride adhesive sheets and using laser ablation to form specific patterns. The high surface area of these electrodes can be utilized to immobilize biomolecules such as aptamers and antibodies. Among the common foodborne pathogens for which the platform was assessed for quantitative detection were *Salmonella typhimurium* and *Listeria monocytogenes*. The outcomes indicated that the system is a quick, accurate, and economical way to monitor food safety and has been successfully used in real-world situations [156]. A similar method was used by Yentongchai et al. to detect *S. enterica* serovar *Typhimurium* using a label-free electrochemical lateral flow immunoassay (LF-eLFIA) combined with NFC technology. The system monitors antigen–antibody interactions without the need for external labeling chemicals by using ferri/ferrocyanide as a redox probe and screen-printed electrodes. The sensor demonstrated a very low LOD of 0.93 CFU mL^−1^ and a wide linear detection range (1–10⁴ CFU mL^−1^). At 4 °C, it held steady for as long as four months. The device functions as a compact LoC and provides a wireless, portable diagnostic solution through the integration of a smartphone app and an NFC-enabled photometric chip. The system’s field readiness was confirmed by validation on raw chicken and eggshell wash samples, which produced good recovery rates (95.6–117%) with RSDs below 3% [157]. Savas et al. used an electrochemical biosensor enhanced with GQDs and nanozyme activity to target the detection of Staphylococcus aureus in phosphate-buffered saline and undiluted milk samples. The technology showed remarkable sensitivity when combined with a smartphone-based electrochemical reader, reaching an LOD of 4 CFU mL^−1^ in milk (Figure 8c). Moreover, the biosensor demonstrated low cross-reactivity to other bacteria, such as *L. monocytogenes*, *St. epidermidis*, *Enterococcus fecium*, and *Streptococcus pneumoniae*, and high specificity for *St. aureus*. These findings demonstrate the sensor’s potential as a useful and dependable instrument for pathogen detection in intricate food matrices [16].

These results show that advances in electrochemical biosensor technologies are enabling portable, user-friendly food analysis tools, mainly when combined with smartphones. Beyond accelerating food safety monitoring, which improves risk management and timely responses to contamination, these methods have substantial potential for practical applications. In the coming years, their integration into routine operations will provide profound benefits to food safety and public health. Although there is a high potential in utilizing electrochemical devices, including LoC technologies, designed to detect pesticides, heavy metals, and foodborne pathogens, their application in practical settings still encounters several difficulties, signifying the need to resolve issues with sensitivity, sustainability, and scalability to achieve wider adoption. The requirement for extremely sensitive, inexpensive, and fully automated sample-to-answer systems, which frequently encounter difficulties in intricate food and environmental matrices, is one of the most significant constraints [35]. The instability of enzyme- or aptamer-based assays in microfluidic environments and interferences from organic residues provide significant challenges for pesticides. Furthermore, the usage of solvents makes it considerably harder to develop devices sustainably. In the case of heavy metals, complex electrodes based on nanomaterials are required to achieve high sensitivity and selectivity for detecting toxic ions like Pb^2+^, Cd^2+^, and Hg^2+^ at trace levels. Nonetheless, the scalable and reproducible integration of such nanostructures remains a key manufacturing challenge [159]. Nucleic acid extraction and amplification are often inhibited in heterogeneous matrices rich in proteins, lipids, and fibers, which makes the detection of foodborne pathogens more difficult. Additional challenges include the need for rapid multiplexing and accurate diagnosis in field settings. The lack of standardized digital procedures, such as secure cloud-based reporting and AI-assisted calibration, can limit widespread adoption. Environmental concerns related to single-use plastics and the need for interdisciplinary training to ensure proper device operation further complicate implementation [160]. Although LoC platforms have great potential for real-time monitoring of pesticides, heavy metals, and pathogens, strong and practical solutions will only be possible by overcoming current challenges in sensitivity, sample preparation, sustainability, and scalability [35].

## 6. From Lab to Field: Bottlenecks and Strategies for Commercializing

Despite impressive lab-scale advances, there are still several bottlenecks in translating smartphone-integrated LoC electrochemical devices into commercial food and agricultural applications. Technical challenges include eliminating signal interference from complex food matrices rich in proteins, lipids, and fibers, which often hinder sensitivity and reproducibility [161]. Long-term sensor stability is another hurdle, as enzyme- or aptamer-based assays typically lose activity during prolonged storage, limiting shelf life and large-scale deployment [162]. Although microfluidics can automate some steps, the integration of filtration, extraction, or nucleic acid amplification into compact devices is still difficult [163]. From an economic perspective, cost control in large-scale manufacturing, particularly for nanomaterials, microfabrication processes, and disposable plastics, remains a key limitation, with only a few low-cost screen-printed electrode platforms reaching near-commercial feasibility [164]. On the regulatory side, approval processes for food safety devices, such as meeting FDA or European Food Safety Authority (EFSA) standards, are lengthy and resource-intensive, requiring extensive validation against gold-standard methods (e.g., LC-MS/MS or PCR) [165,166]. Finally, user adoption thresholds such as ease of operation, requirement for interdisciplinary training, and reliability under resource-limited conditions (e.g., smallholder farms without stable power or internet access) remain significant obstacles [167]. A few case studies highlight both progress and persistent gaps: for example, COVID-19 LoC diagnostic kits rapidly gained emergency approval, showing the potential for fast regulatory pathways under urgent demand [168], whereas most food-contaminant sensors still remain confined to pilot studies. Addressing these barriers through standardization, cost-efficient materials, modular microfluidic sample preparation, and adaptive design for diverse global agricultural contexts will be essential for successful commercialization.

Several practical strategies are emerging to overcome the main bottlenecks in scaling smartphone-integrated LoC electrochemical sensors towards real-world deployment. To prevent matrix interference and sensor fouling, antifouling surface modifications such as zwitterionic peptide or polymer coatings have demonstrated improved performance in complex food environments [169]. Streamlined on-chip sample preparation is achievable by implementing magnetic bead–based extraction and pathogen isolation modules that automate sample-in-answer workflows [170]. Power and cost constraints can be reduced using battery-free NFC-powered devices capable of harvesting energy from smartphones, while scalable, low-cost fabrication via lab-on-printed circuit boards (PCBs) or screen-printing techniques significantly cuts production costs [171]. To improve long-term stability, adopting enzyme-free biorecognition elements like aptamers or nanobodies minimizes degradation risks and supports field reliability [172]. Addressing regulatory and usability hurdles, early adoption of app-guided, POC testing-style validation protocols coupled with cloud integration can streamline standard compliance and user training requirements [173]. Finally, using sustainable, paper-based microfluidic platforms helps meet environmental goals and simplifies device logistics in resource-limited settings [174]. In addition to existing strategies, multiplexed microfluidic architectures and CRISPR (clustered regularly interspaced short palindromic repeats)-based assays are being explored to enable simultaneous detection of multiple contaminants in a single test. For example, a one-pot microfluidic biosensor combining CRISPR/Cas12a (CRISPR-associated protein 12a) with recombinase-aided amplification enabled detection of seven common foodborne pathogens within an hour, using a smartphone for readout [12,175]. Furthermore, the use of edge AI algorithms on smartphones allows rapid, offline data processing—crucial for deployment in rural or low-connectivity settings—by running machine learning inferences directly on-device without relying on cloud connectivity [176]. Together, these advances complement current approaches and further strengthen the transitional pathway from laboratory prototypes to field-ready commercialization.

## 7. Conclusions and Future Perspectives

Due to recent developments in microfluidics, nanomaterials, and mobile technology, smartphone-integrated electrochemical devices, including LoC biosensors, are modern high-potential instruments for tracking pollutants in food and agriculture. These devices provide real-time, on-site detection of a variety of contaminants, including infections, pesticides, heavy metals, and mycotoxins, directly at farms, markets, and food processing plants by combining high sensitivity, portability, and user-friendliness. These devices not only minimize sample preparation, expense, and reliance on laboratory infrastructure but also provide precise, quantitative results by integrating cellphones with electrochemical sensing platforms.

Nevertheless, several obstacles can be identified against their general implementation. When they are integrated with various smartphone models, compatibility problems can lead to changes in the device’s performance. Furthermore, maintaining sensitivity and reproducibility with small sample sizes is not easy. Restrictions, including battery life, security of wireless connectivity, data standardization, and the requirement for user training, continue to be significant obstacles. These challenges should be addressed before electrochemical platforms can be widely adopted in the real world. Despite these practical obstacles, these technologies remarkably improve public health, environmental sustainability, and food safety by facilitating quicker reaction times and better risk management. Their contribution to global food safety will be further reinforced in the future by advancements in sensitivity, multiplexing, strength, and validity. On the one hand, large-scale, real-time surveillance networks will be supported by IoT integration. On the other hand, advances in AI and ML can significantly promote data processing and prediction models. Important next stages include constructing eco-friendly, biodegradable materials, increasing the shelf life of sensors, and standardizing procedures for regulatory approval. These technologies will also become increasingly more adaptable and significant in assuring food security and public health if they are extended to handle new contaminants and modified for wider environmental and therapeutic applications.

Future biosensor technology development will be heavily reliant on interdisciplinary co-operation. Designing high-selectivity recognition components, including enzymes, antibodies, and aptamers, as well as developing innovative nanomaterials for improved sensitivity and specificity, will depend on developments in chemistry and biology. The creation of flexible, miniature electrodes and energy-efficient circuits can guarantee excellent performance in wearable and portable devices for electronics and materials research. Real-time monitoring, remote diagnostics, and predictive analytics utilizing AI-driven algorithms, ML models, and IoT-based platforms are simultaneously made possible by software engineering and data science. Also, the practical framework for monitoring soil and water quality and detecting pesticides, heavy metals, and foodborne pathogens at the field level is provided by agricultural and environmental sciences. The translation of these technologies into healthcare applications will also be supported by partnerships with the biomedical sciences, which will guarantee biocompatibility and ease clinical validation. In summary, the development of portable, intelligent, and adaptable next-generation biosensors that can be used in food safety, agriculture, healthcare, and environmental monitoring will be accelerated by these interdisciplinary contributions.

## Data Availability

No new data were created or analyzed in this study. Data sharing is not applicable to this article.

## Abbreviations

The following abbreviations are used in this manuscript:

AFB1	Aflatoxin B1
AIEgen	Aggregation-Induced Emission Generator
AI	Artificial Intelligence
ASSURED	Affordable, Sensitive, Specific, User-friendly, Rapid and Robust, Equipment-free, Deliverable to End-Users
AuNPs	Gold Nanoparticles
CA	Chronoamperometry
CBA	Carbaryl
CNN	Convolutional Neural Network
CPUs	Central Processing Units
CV	Cyclic Voltammetry
COF/MB@MnO_2_	Covalent Organic Framework/Methylene Blue@Manganese Dioxide
CRISPR	Clustered Regularly Interspaced Short Palindromic Repeats
DPV	Differential Pulse Voltammetry
EDCs	Endocrine-Disrupting Chemicals
EIS	Electrochemical Impedance Spectroscopy
ELISA	Enzyme-Linked Immunosorbent Assay
EµPAD	Electrochemical Microfluidic Paper-based Analytical Device
FETs	Field-Effect Transistors
FNT	Fenitrothion
GLE	Gold Leaf Electrode
GPUs	Graphics Processing Units
IC_50_	Half Maximal Inhibitory Concentration
IoT	Internet of Things
LC-MS/MS	Liquid Chromatography–Tandem Mass Spectrometry
LoC	Lab-on-a-Chip
LOD	Limit of Detection
LoRaWAN	Long Range Wide Area Network
ISE	Ion Selective Electrode
LSV	Linear Sweep Voltammetry
MCU	Microcontroller Unit
μMISPE-MS	Micro Molecularly Imprinted Solid-Phase Extraction—Mass Spectrometry
μTAS	Micro Total Analysis System
MIP	Molecularly Imprinted Polymer
ML	Machine Learning
MOF	Metal–Organic Framework
MRL	Maximum Residue Limit
MRS	Magnetic Relaxation Switch
NFC	Near Field Communication
NMR	Nuclear Magnetic Resonance
OP	Organophosphorus Pesticide
OWLS	Optical Waveguide Lightmode Spectroscopy
PCBs	Printed Circuit Boards
PCR	Polymerase Chain Reaction
PDMS	Polydimethylsiloxane
PET	Polyethylene Terephthalate
PFAS	Per- and Polyfluoroalkyl Substances
PFOS	Perfluorooctane Sulfonate
(P)GO	(Porous) Graphene Oxide
PPCPs	Pharmaceuticals and Personal Care Products
QCM	Quartz Crystal Microbalance
RCA	Rolling Circle Amplification
rGO	Reduced Graphene Oxide
RhizoChip	Rhizosphere on a Chip
RSD	Relative Standard Deviation
SBC	Single Board Computer
SNR	Signal-to-Noise Ratio
SPCE	Screen-Printed Carbon Electrode
SPR	Surface Plasmon Resonance
SWV	Square Wave Voltammetry
Wi-Fi	Wireless Fidelity
